# Saliva Dysfunction and Oral Microbial Changes among Systemic Lupus Erythematosus Patients with Dental Caries

**DOI:** 10.1155/2018/8364042

**Published:** 2018-04-02

**Authors:** Lijie Yang, Jing Wang, Yan Xiao, Xi Wang, Qiang Sun, Junlan Shang, Yulin Zhao

**Affiliations:** ^1^Department of Rhinology, The First Affiliated Hospital of Zhengzhou University, Zhengzhou, China; ^2^Department of Stomatology, The First Affiliated Hospital of Zhengzhou University, Zhengzhou, China

## Abstract

Systemic lupus erythematosus (SLE) is a chronic autoimmune and inflammatory disease affecting multiorgans of human body. Independent studies show that SLE patients had higher caries prevalence compared to non-SLE individuals. However, the underlying mechanisms remain unclear. In present study, we enrolled SLE patients to explore potential factors contributing to the susceptibility of SLE patients to dental caries (such as oral hygiene, salivary function, and oral microbial community). Dental examination confirmed SLE patients were more vulnerable to caries. Although subjects in both groups announced similar oral hygiene habits, more dental plaque was found on tooth surfaces of SLE patents as revealed by plaque index. In addition, the salivary function was impaired in SLE group as salivary flow rate, buffering capacity, and pH were lower among SLE subjects compared to healthy controls. Importantly, disturbed microbial community with lower richness and diversity was observed in SLE group, as well as disequilibrium between acidogenic/aciduric pathogens and alkali-generating commensal bacteria. Our data suggest that SLE increases patients' sensitivity to dental caries through imposing stress to both host and oral microbes.

## 1. Introduction

Systemic lupus erythematosus (SLE) is a chronic disease affecting several organs of the human body. It is a heterogeneous disease with autoimmune origin and is characterized by the presence of autoantibodies against nuclear antigens [1]. The worldwide prevalence of SLE ranges between 12 and 50 per 100,000, varying between locations and ethnicities [2]. Women, especially those in their 40s, are more vulnerable than men [3].

Oral manifestations have been found frequently in SLE patients, including recurrent infections or mouth ulcers, severe gingivitis, temporomandibular joint disorder, osteonecrosis of the mandible, hyposalivation (decrease of salivary flow), and excessive dental caries [4–6]. Among these implications, higher dental caries prevalence among SLE patients has been confirmed by several independent clinical investigations [5, 6]. Dental caries is a chronic infection initiated by microbes in the human mouth. Through metabolizing carbohydrates, oral microorganisms produce both organic acid and exopolysaccharides (EPS). With the help of EPS-rich matrix, the organic acid maintains a low pH microenvironment adjacent to the tooth surface, which causes the demineralization of tooth hard tissue and, ultimately, the caries [7]. During this process, saliva around the teeth plays caries-preventive functions by its flushing and neutralizing effects. Generally speaking, high salivary flow rate, and buffer capacity decrease the caries risk [8].

Although some investigations have revealed the association between SLE and dental caries, the study aimed at exploring reasons why SLE patients are more vulnerable to dental caries is still in its infancy, and the existent findings seem to be controversial. For example, Loyola Rodriguez et al. (2016) found that* Streptococcus mutans* and* Streptococcus sobrinus* species were enriched in SLE patients' salivary microbiome, while a decrease in salivary flow rate, pH, and buffer capacity was observed compared to healthy subjects [6]. However, de Araujo Navas found no significant differences in frequency and abundance of* Candida *spp.,* Staphylococcus* spp.,* Enterobacteria,* and* Pseudomonas* spp. between SLE patients and healthy individuals [9]. Apart from disagreement among these investigations, it is noteworthy that microbial studies listed above focused on specific cariogenic microbes. However, dental plaque is polymicrobial disease, and it is the microbial community rather than the presence/absence of specific bacteria that determines whether caries occurs or not [10, 11]. Thus, a comprehensive understanding of the microbial diversity, composition, and structure of oral microbial community in SLE patients will provide insightful clues for the higher caries risk of these sufferers.

Considering saliva function and oral microbial community are both involved in the pathogenesis of dental caries, we hypothesized that SLE might increase the susceptibility of SLE patients to dental caries through impairing saliva function and introducing disequilibrium into oral microbial community. To test our hypothesis, treatment-naïve SLE patients and healthy controls were recruited in present clinic study. The caries frequency was checked; then oral hygiene habit, saliva variables (i.e., flow, pH, and buffer capacity), and characteristics of salivary microbial community (i.e., richness, diversity, microbial structure, and disequilibrium between acidogenic pathogens and alkali-generating commensal bacteria) were investigated.

## 2. Material and Methods

**Figure 1:**
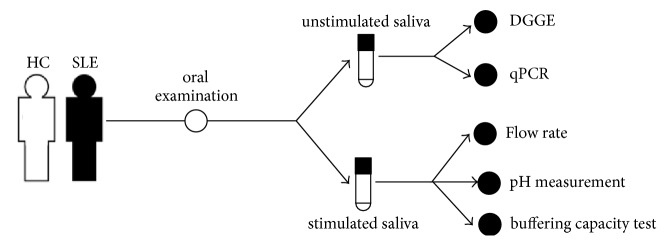
Study design. SLE: systemic lupus erythematosus; HC: healthy control.

This study is conducted in accordance with the Declaration of Helsinki (1964) and is approved by the ethical committee of the First Affiliated Hospital of Zhengzhou University. Informed and voluntary written consent from subjects were obtained. The overall design of present study was shown in Figure 1.

*Subject Enrollment*. Patients were enrolled when they visited the hospital searching for medical help due to SLE. The diagnosis was performed by a rheumatologist using the criteria of the American College of Rheumatology [12] first, and only these did not receive anti-SLE treatment before being invited to participate in this study to exclude the influence of medication on saliva function and microbiome. Then these SLE treatment-naïve subjects (*N* = 347) completed a health questionnaire regarding their systemic health. Exclusion criteria included pregnancy, occurrence of other systemic diseases (e.g., diabetes mellitus, systematic infection), and receiving antidepressants treatment in the last 3 months or taking antibiotics/antifungal drugs in the past 1 week. Following the systemic health questionnaire, the oral health was screened by a dentist. Noninclusion criteria included detectable oral diseases other than dental caries, harmful behaviors (e.g., smoking, alcoholism), and use of removable dentures. Finally, 20 SLE patients meet the inclusion and exclusion criteria and were included in present clinical study, and majority of enrolled subjects (2 out of 18) were women. Moreover, another group of age- and sex-matched healthy people (*N* = 20) were recruited as controls.

*Oral Hygiene Survey*. The frequency of tooth brush per day, use of fluoride products, and artificial saliva of each subject were reported and recorded.

*Caries Examination*. Dental examination was carried out by a dentist with the WHO criteria for the diagnosis and coding of caries [13]. The WHO TRS-621 C-version periodontal probe was used to confirm visual evidence of caries. A carious lesion was recorded present when a lesion had a cavity, had undermined enamel, had decayed softened floor or wall, or felt soft or leathery on probing. A dental restoration with secondary decay was also recorded as a caries lesion. The dental caries were scored by the DMFT index counting the number of decayed (D), missing (due to caries only, M), and filled (F) teeth.

*Plaque Index Assay*. The plaque index (PI) was recorded as described previously [14]. PI index records both soft debris and mineralized deposits on tooth surfaces. Selected teeth (including #16, #12, #24, #32, #36, and #44) were examined, and each of the four surfaces of the tooth (buccal, lingual, mesial, and distal) is given a score ranging from 0 to 3. The scores from the four areas of the tooth are added and divided by four in order to give the plaque index for the tooth, and the PI for each person is the average PI of all selected teeth.

*Saliva Collection for Microbial Assay*. Volunteers were asked to not eat or drink for 8 hours and to refrain from oral hygiene (such as brushing or flossing of teeth) for 12 h before sampling, and each subject was instructed to expectorate about 5 ml spontaneous, unstimulated whole saliva into a sterile cryogenic vial put on ice (Corning, NY, USA) in the morning [15]. Specimens were transported to the laboratory within 2 h on ice and stored at −80°C before further analysis.

*Saliva Flow Rate and Buffer Capacity Analysis*. The saliva flow rate and buffer capacity assay were conducted 1 h after microbial sampling. After resting for 5 minutes, volunteers chewed a piece of paraffin wax (30 seconds) and expectorated into a 50 mL cryogenic vial on ice within 5 minutes [15]. The salivary pH was measured with a pH meter (Metrohm 632, Herisau, Switzerland), and buffering capacity was determined by the dip-slide technique following the manufacturer's instructions (CRT bacteria, Ivo-clar Vivadent, Germany).

*DNA Extraction and PCR-DGGE*. About 1 ml of saliva was used for genomic DNA isolation with QIAamp DNA micro Kit (Qiagen, Valencia, CA, USA), and an extra lysozyme treatment was added to lyse cell wall as described by Wang et al. [16]. The concentration and purity of the extracted gDNA was measured, and DNA samples were put at −20°C until use.

The universal primers targeting the V4-V5 hypervariable region (~300 bp) of the 16S rRNA gene locus were used in the PCR amplification, and the sequence of primers was Bac1 (5′-CGCCCGGGGCGCGCCCCGGGCGGGGCGGGGGCACGGGGGGACTACGT-GCCAGCAGCC-3′) and Bac2 (5′-GGACTACCAGGGTATCTACTAATCC-3′) [17]. The volume of PCR reaction mix was 50 *μ*L, containing 100 ng genomic DNA, 40 pmol of forward and reverse primer, 200 *μ*mol/L dNTPs, 4 mmol/L MgCl_2_, 5 *μ*L 10x PCR buffer, and 2.5 U Taq DNA polymerase (Invitrogen, California, USA) [18]. Cycling conditions were 94°C for 3 min, followed by 30 cycles of 94°C for 1 min, 56°C for 1 min, and 72°C for 2 min, with a final extension period of 5 min at 72°C [19]. The resulting amplicons were confirmed by electrophoresis in 1% agarose gels.

Polyacrylamide gels (8% (w/v)) were prepared with a denaturing urea/formamide gradient between 40% (containing 2.8 mol/L urea and 16% (v/v) formamide) and 60% (containing 4.2 mol/L urea and 24% (v/v) formamide). About 300 ng of amplicons was loaded into each well. The gels with PCR products were submerged in 1x TAE buffer, and the loaded DNA fragments were separated by electrophoresis at 58°C using a fixed voltage of 60 V in the Bio-Rad DCode System for 17 h (Bio-Rad Laboratories, Inc., Hercules, CA). Immediately after electrophoresis, gels were rinsed once, stained using 0.5 *μ*g/mL ethidium bromides prepared with 1x TAE buffer for 15 min, and destained in 1x TAE buffer for 10 min. DGGE images were captured through Molecular Imager Gel Documentation system (Bio-Rad Laboratories).

*qPCR Analysis of Oral Samples*.* S. mutans*,* S. sobrinus*,* S. sanguinis*,* S. gordonii*,* Actinomyces naeslundii,* and all bacterial counts were quantified using qPCR as previously described [20]. Primers and probes used were listed in Table S1. Each sample was examined in triplicate. The quantitative amplification condition using Bio-Rad CFX96 system, and the MIQE (Minimum Information for Publication of Quantitative Real-Time PCR Experiments) guidelines were followed for quality control of the data generated and for data analysis.

*Statistical Analysis*. Mann-Whitney *U* test was used to compare age, DMFT scores, tooth brushing frequency, plaque index, and qPCR data between groups.

DGGE images were normalized and analyzed with Quantity One Software (Bio-Rad Laboratories, Hercules, CA, USA). Bands detected from each sample were taken as microbial richness, and alpha diversity comparison was based on Shannon index taking both gel tracks and band intensity into consideration [21]. The unweighted pair group method with arithmetic means (UPGMA) was used to construct dendrogram to compare banding patterns, and principal component analysis (PCA) was performed using SPSS version 13.0 (Statistical Package for the Social Science, SPSS Inc., Chicago, IL, USA) [16].

For intergroup comparison of salivary parameters, richness, and diversity, the quantitative data was first analyzed using Levene's test for homogeneity of variances to assess the equality of variances. One-way ANOVA with Dunnett *t*-test was then used.

## 3. Results and Discussion

### 3.1. Dental Examination Reveals a Pressing Need of Caries Interventions for SLE Patients

Although independent clinical investigations indicate the higher prevalence of dental caries in SLE patients, the factors contributing to the susceptibility of SLE sufferers to tooth decay was still unclear. In present study, SLE patients (*N* = 20) and healthy volunteers (*N* = 20) were enrolled to investigate their dental health, oral hygiene, saliva function, and oral microbiome, all of which are highly associated with caries pathogenicity [8, 10, 22].

The mean age of enrolled SLE patients was 42.4 (Table 1), which was consistent with previous report that women aged 40–49 years are more prone to influenced by SLE [23]. The potential reasons for higher prevalence of SLE in individuals at their 40s might be related to aging of the population, better access to healthcare, and increased serologic monitoring, or reflecting the unique pathophysiology of disease [23]. Increased exposure of the aging population to numerous drugs, many of which can cause a lupus flare, may be another explanation [23].

Before sampling, the dental health was checked first. The caries prevalence of SLE was 100% in our pilot, and enrolled SLE patients had at least 6 teeth infected by dental caries with average DMFT higher than 11 (Table 1). Moreover, the tooth surface most frequently influenced by dental caries was occlusal surface, followed by proximal surfaces. According to the 3rd national oral health epidemiological survey of China, the caries prevalence of Chinese is 88.1% among these aged within 35~44, and the average DMFT is ~4.5 [24]. Clearly, SLE patients had more heavy caries burden, and dental intervention are necessary to improve their life quality.

### 3.2. Poor Dental Hygiene Is Common among SLE Patients

The clinical investigation carried out in Chinese population in present study confirmed that SLE patients are vulnerable to dental caries, as did by other independent study carried out in other countries [5, 6]. To reveal the deeper mechanisms involved in SLE patients' susceptibility, we further did an oral hygiene survey by questionnaire and oral examination, as good oral hygiene is important to caries prevention [22, 25]. None of the subjects in both groups had clinic use of fluoride products (such as fluoride varnishes) and artificial saliva, as well as fluoride or antibiotics mouth rinse (Table 2). However, all of them used fluoride toothpaste in daily life (Table 2). The most effective way to remove dental plaque is tooth brushing. Although subjects in both groups announced that they brush teeth with similar frequency (i.e., twice per day) and with toothpaste containing the same anticaries agent (fluoride), the plaque control result was significantly different between groups as the plaque index (PI) of SLE sufferers was twice higher than that of healthy control (1.78 versus 0.84, *p* < 0.05, Table 2), suggesting that tooth surfaces of SLE patents were covered by more dental plaque. The potential reasons might be that poor self-care ability and discomfort caused by SLE reduced the quality of tooth brushing. Since dental plaque is the initiative factor of caries, we speculated that poor plaque control was among the reasons why SLE patients were susceptible to dental caries.

### 3.3. The Salivary Function of SLE Patients Is Impaired

Saliva plays a significant role in the prevention of dental caries, such as antibacterial activity, flushing the oral cavity, removing food particles and debris, and chemically maintaining an environment rich in acid-buffering agents [8]. Considering the crucial roles of saliva in dental caries, we tested flow rate, pH, and buffering capacity of stimulated saliva in present study to see if the salivary function of SLE patients was affected, and the result was shown in Table 3. In SLE group, the salivary flow rate was 0.78 ± 0.05 ml/min, much lower than healthy individuals (1.32 ± 0.09 ml/min), reflecting a dysfunction in the salivary glands of the disease group. Several other studies also pointed that hyposalivation was common in SLE patients [6, 26, 27], although the salivary flow varied due to different protocols and sample size. Besides, the activity of the disease, age, and the drugs used were associated with hyposalivation [26], which could also introduce variations among these study. The mean salivary pH was also lower in the SLE group (6.74 ± 0.05) than in the control group (7.03 ± 0.05), and there was statistically significant difference between the two groups. Moreover, our data demonstrated that subjects with high saliva buffering capacity decreased in SLE group. In general, the higher the flow rate, the faster the clearance and the higher the buffering capacity and thus the lesser microbial attack and caries activity [8]. The decreased salivary flow rate and buffering capacity definitely had a detrimental effect on caries. In relation to pH, it has been well documented that the dissolution of enamel occurs when the pH falls below critical pH (i.e., 5.5), so the values obtained in the study are not adequate to cause demineralization of tooth hard tissues.

### 3.4. The SLE Patients Harbor a Disturbed Oral Microbial Community

**Figure 2:**
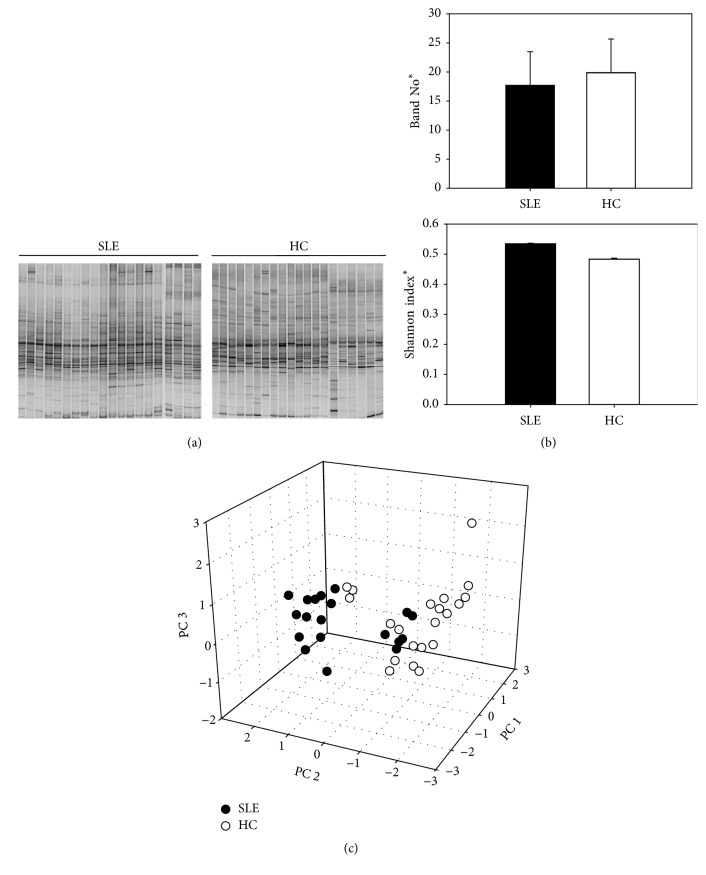
Salivary microbial community differences between SLE group and health control (HC). (a) DGGE images; (b) richness (band number) and *α*-diversity (Shannon index) comparison; (c) PCA analysis of DGGE profiles. SLE: systemic lupus erythematosus; Band No.: band number. *∗* indicates *p* < 0.05.

For decades, mutans streptococci, especially* S. mutans, *were regarded as the specific etiology of dental caries. However, this mutans-centric paradigm was challenged when other acid-producing oral microorganisms were isolated from carious lesions and found to be strongly associated with the disease. Thus, a new point emerged in which complex bacterial communities appeared to be associated with the disease other than specific pathogens. Considering the crucial role of oral microbial community in the pathogenesis of dental caries, we applied DGGE method to investigate if SLE imposed stress to the oral microbial community, caused symbiosis, and ultimately induced caries initiation. DGGE profiles showed that shared bacteria were found among samples; however, interindividual variations could be also observed among samples even these from the same group (Figure 2(a)). Some interesting results were obtained from the DGGE analysis. First, we detected 60 bands from the DGGE images, suggesting that there were 60 bacterial species in these saliva samples. Since only taxa with relative abundance higher than 1% could be detected by DGGE, we admitted that present data underestimated the richness of salivary bacteriome since rare taxa was not taken into account. Second, microbial richness was estimated using band number, and Shannon index was used to compare the alpha diversity. The band number of healthy group was about 20 (19.80 ± 2.34) on average while less bands was picked from SLE (17.47 ± 2.30), and the difference was statistically significant (*p* < 0.05, Figure 2(b)). Regarding the *α*-diversity, lower diversity was observed in SLE group compared to that in healthy controls (*p* < 0.05, Figure 2(b)). Third, to see if the overall salivary microbial community of SLE patients was distinct from that of healthy persons, DGGE images were analyzed using clustering analysis and principle component analysis (PCA). The PCA grouped the 40 samples into two separate clusters as shown in Figure 2(c), indicating distinct microbial community between SLE and healthy control. The stress imposed by SLE seems to greatly influence the salivary microbial community as showing in DGGE assay that overall microbial structure, as well as the community richness and evenness, changed significantly compared to healthy control. The decreased richness and diversity could be a very important contributor to disease progression because, from an ecological perspective, the reduced biodiversity probably create favorable environment for prosperity of pathogenic strain as interspecies antagonisms could be impaired or diminished [28].

**Figure 3:**
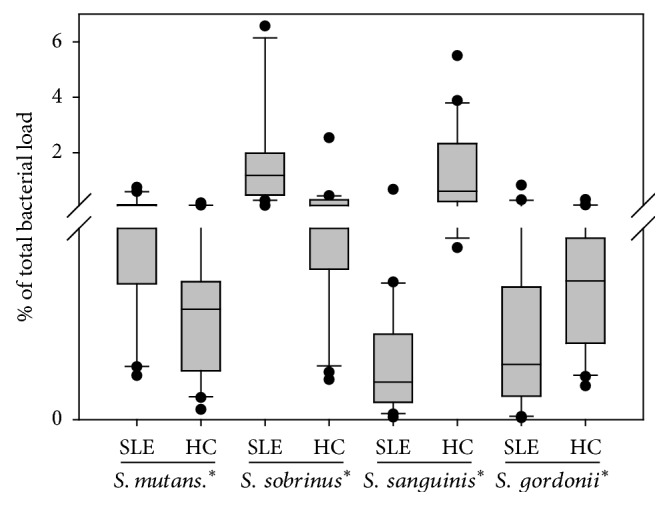
Comparison of relative abundance of acidogenic bacteria (*S. mutans* and* S. sobrinu*s) and ammonia producing bacteria (*S. sanguinis* and* S. gordonii*). SLE: systemic lupus erythematosus; HC: healthy control; *∗* indicates *p* < 0.05.

The DGGE assay shows that oral microbiome of SLE changed a lot compared to healthy control without explaining how it. To give a detailed example how SLE changed the oral microbial community, we further used qPCR to determine the relative abundance of some specific taxa including acidogenic bacteria (*S. mutans* and* S. sobrinu*s) and ammonia producing bacteria (*S. sanguinis* and* S. gordonii*). These four bacteria were chosen since the pH drop caused by organic acid produced by acidic and aciduric bacteria is among the most important virulence of cariogenic bacteria while* S. sanguinis* and* S. gordonii* in the biofilm can also generate alkali to antagonize the pH drop through arginine metabolism [29, 30]. As shown in Figure 3, the proportion of* S. sanguinis* and* S. gordonii *in the saliva of SLE patients was statistically higher than that of control group (*p* < 0.05). However,* S. sanguinis* and* S. gordonii *showed a decreasing tendency in SLE group (*p* > 0.05). These data suggested that microbial disequilibrium between acidogenic/aciduric pathogens and alkali-generating commensal bacteria colonized in the oral cavity might be involved in the caries risk of SLE patients.

In conclusion, our data further confirmed that SLE patients were more vulnerable to caries infection. The poor plaque control, dysfunction of saliva gland, and disturbed microbial community with microbial disequilibrium increase patients' sensitivity to dental caries. However, we admit that the conclusion drawn from present study should be taken carefully due to the small sample size, and more investigations are needed to confirm it. Furthermore, studies are necessary to reveal through which pathways SLE plays its influence on saliva function and oral microbial community.

## Figures and Tables

**Table 1:** Clinical features and dental examination of enrolled subjects.

	SLE (*N* = 20)	HC (*N* = 20)	Statistical Method
Age (Year)	42.4 ± 5.8	43.0 ± 6.0	Mann-Whitney *U* test
Sex (M/F)	2/18	2/18	Null
DMFT^*∗*^	11.05 ± 5.12	0.00	Mann-Whitney *U* test

*Abbreviation*. SLE-systemic lupus erythematosus; HC-Healthy control; N-Number of subject; F-Female; M-Male; DMFT-Decay, Missing, Filling tooth; *∗* indicates *p* < 0.05.

**Table 2:** Oral hygiene survey of enrolled subjects.

	SLE (*N* = 20)	HC (*N* = 20)	Statistical Method
F (tooth brushing)	2.15 ± 0.48	2.10 ± 0.55	Mann-Whitney *U* test
N (Fluorides toothpaste)	20	20	NULL
N (artificial saliva)	0	0	NULL
PI^*∗*^	1.78 ± 0.32	0.84 ± 0.35	Mann-Whitney *U* test

*Abbreviation*. SLE-systemic lupus erythematosus; HC-Healthy control; N-Number of subject; F (tooth brushing)-the frequency of tooth brushing per day; N (Fluorides toothpaste)- number of subjects using fluorides toothpaste; N (artificial saliva)- number of subjects using artificial saliva. PI-plaque index; *∗* indicates *p* < 0.05.

**Table 3:** Comparison of salivary factors involved in dental caries progression between SLE and control group.

	Stimulated saliva flow rate (ml/min)^*∗*^	Saliva pH^*∗*^	Buffering capacity No. of patients		
			Low	Medium	High
SLE	0.78 ± 0.05	6.74 ± 0.05 7.03 ± 0.05	2	11	7
HC	1.32 ± 0.09		2	3	15

*Abbreviation*. SLE-systemic lupus erythematosus; HC-Healthy control; *∗* indicates *p* < 0.05.

## Supplementary Materials

Supplementary Materials Table S1: primers and probes used in qPCR.

## References

[1] Tsokos G. C. (2011). Systemic lupus erythematosus. *The New England Journal of Medicine*.

[2] Pons-Estel G. J., Alarcón G. S., Scofield L., Reinlib L., Cooper G. S. (2010). Understanding the epidemiology and progression of systemic lupus erythematosus. *Seminars in Arthritis and Rheumatism*.

[3] Danchenko N., Satia J. A., Anthony M. S. (2006). Epidemiology of systemic lupus erythematosus: a comparison of worldwide disease burden. *Lupus*.

[4] Urman J. D., Lowenstein M. B., Abeles M., Weinstein A. (1978). Oral mucosal ulceration in systemic lupus erythematosus. *Arthritis & Rheumatism*.

[5] Pascual-Ramos V., Hernández-Hernández C., Soto-Rojas A. E. (2006). Association between dental caries and pneumonia in patients with systemic lupus erythematosus. *The Journal of Rheumatology*.

[6] Loyola Rodriguez J. P., Galvan Torres L. J., Martinez Martinez R. E. (2016). Frequency of dental caries in active and inactive systemic lupus erythematous patients: Salivary and bacterial factors. *Lupus*.

[7] Xiao J., Klein M. I., Falsetta M. L. (2012). The exopolysaccharide matrix modulates the interaction between 3D architecture and virulence of a mixed-species oral biofilm. *PLoS Pathogens*.

[8] Wang R. k., Zhou X. (2016). Saliva and Dental Caries. *Dental Caries: Principles and Management*.

[9] de Araujo Navas E. A., Sato E. I., Pereira D. F. (2012). Oral microbial colonization in patients with systemic lupus erythematous: correlation with treatment and disease activity. *Lupus*.

[10] Selwitz R. H., Ismail A. I., Pitts N. B. (2007). Dental caries. *The Lancet*.

[11] Takahashi N., Nyvad B. (2011). The role of bacteria in the caries process: ecological perspectives. *Journal of Dental Research*.

[12] Pons-Estel G. J., Wojdyla D., McGwin G. (2014). The American College of Rheumatology and the Systemic Lupus International Collaborating Clinics Classification criteria for systemic lupus erythematosus in two multiethnic cohorts: a commentary. *Lupus*.

[13] Clerehugh V. (1989). Oral health surveys: Basic methods, 3rd edition. *Journal of Dentistry*.

[14] Silness J., Löe H. (1964). Periodontal disease in pregnancy. II. correlation between oral hygiene and periodontal condtion. *Acta Odontologica Scandinavica*.

[15] Zhang J., Liu H., Liang X. (2015). Investigation of salivary function and oral microbiota of radiation caries-free people with nasopharyngeal carcinoma. *PLoS ONE*.

[16] Wang K., Miao T., Lu W. (2015). Analysis of oral microbial community and Th17-associated cytokines in saliva of patients with oral lichen planus. *Microbiology and Immunology*.

[17] Rupf S., Merle K., Eschrich K. (1999). Quantification of Bacteria in Oral Samples by Competitive Polymerase Chain Reaction. *Journal of Dental Research*.

[18] Wang R.-K., He X.-S., Hu W. (2011). Analysis of interspecies adherence of oral bacteria using a membrane binding assay coupled with polymerase chain reactiondenaturing gradient gel electrophoresis profiling. *International Journal of Oral Science*.

[19] Wang R., Kaplan A., Guo L. (2012). The Influence of Iron Availability on Human Salivary Microbial Community Composition. *Microbial Ecology*.

[20] Yoshida A., Suzuki N., Nakano Y., Kawada M., Oho T., Koga T. (2003). Development of a 5′ nuclease-based real-time PCR assay for quantitative detection of cariogenic dental pathogens Streptococcus mutans and Streptococcus sobrinus. *Journal of Clinical Microbiology*.

[21] Boon N., Windt W., Verstraete W. (2002). Evaluation of nested PCR-DGGE (denaturing gradient gel electrophoresis) with group-specific 16S rRNA primers for the analysis of bacterial communities from different wastewater treatment plants. *FEMS Microbiology Ecology*.

[22] Liang J., Wu B., Plassman B., Bennett J. M., Beck J. (2014). Social stratification, oral hygiene, and trajectories of dental caries among old americans. *Journal of Aging and Health*.

[23] Jarukitsopa S., Hoganson D. D., Crowson C. S. (2015). Epidemiology of systemic lupus erythematosus and cutaneous lupus erythematosus in a predominantly white population in the United States. *Arthritis Care & Research*.

[24] Qi X. (2008). *The Third National Sampling Epidemiological Survey on Oral Health*.

[25] Bellini H. T., Arneberg P., Von Der Fehr F. R. (1981). Oral hygiene and caries: A review. *Acta Odontologica Scandinavica*.

[26] Leite C. A., Galera M. F., Espinosa M. M. (2015). Prevalence of hyposalivation in patients with systemic lupus erythematosus in a Brazilian subpopulation. *International Journal of Rheumatology*.

[27] Ben-Aryeh H., Gordon N., Szargel R., Toubi E., Laufer D. (1993). Whole saliva in systemic lupus erythematosus patients. *Oral Surgery, Oral Medicine, Oral Pathology, Oral Radiology, and Endodontology*.

[28] Chapin F. S., Zavaleta E. S., Eviner V. T. (2000). Consequences of changing biodiversity. *Nature*.

[29] Liu Y.-L., Nascimento M., Burne R. A. (2012). Progress toward understanding the contribution of alkali generation in dental biofilms to inhibition of dental caries. *International Journal of Oral Science*.

[30] Nascimento M. M., Burne R. A. (2014). Caries Prevention by Arginine Metabolism in Oral Biofilms: Translating Science into Clinical Success. *Current Oral Health Reports*.

