# Supplementary material for: Saliva Dysfunction and Oral Microbial Changes among Systemic Lupus Erythematosus Patients with Dental Caries
Source: Biomed Res Int. 2018 Apr 2;2018:8364042. doi: 10.1155/2018/8364042 (PMC5902118; doi:10.1155/2018/8364042)
Supplement: Supplementary Materials — Table S1: primers and probes used in qPCR. [file 8364042.f1.docx]

| Bacteria | Target gene | Primers/probes |
| --- | --- | --- |
| *S. mutans*  *S.sanguinius* | *gtfB*  *gtfP* | F: 5’-GCCTACAGCTCAGAGATGCTATTCT-3’  R: 5’-GCCATACACCACTCATGAATTGA-3’  P: 5’-FAM-TGGAAATGACGGTCGCCGTTATGAA-TAMRA-3’  F: 5’-GAGCGGATGGCCAATTATATCT-3’  R: 5’-CCGGATGATGTCGGCAATA-3’  P: 5’-FAM-TGTTCGGGCTCATGATA-TAMRA-3’ |
| *S. gordonii* | *arcA* | F: 5’-GGTGTTGTTTGACCCGTTCAG-3’  R: 5’-AGTCCATCCCACGAGCACAG-3’  P: 5’-FAM-AACCTTGACCCGCTCATTACCAGCTAGTATG-TAMRA-3’ |
| *S. sobrinus* | *gtfT* | F: 5’-TTCAAAGCCAAGACCAAGCTAGT-3′  R: 5’-CCAGCCTGAGATTCAGCTTGT-3′  P: 5’-FAM-CCTGCTCCAGCGACAAAGGCAGC-TAMRA-3′ |
| Universal | 16S rRNA | F: 5’-CGCAGAAGGTGAAAGTCCTGTAT-3’  R: 5’-TGTGACGGGCGGTGTGTA-3’  P: 5’-FAM-CACGGTGAATACGTTCCCGGGC-TAMRA-3’ |

**Table S1. Primers and probes used in qPCR**
